# MHD Free convection flows of Jeffrey fluid with Prabhakar-like fractional model subject to generalized thermal transport

**DOI:** 10.1038/s41598-023-36436-2

**Published:** 2023-06-07

**Authors:** Imran Siddique, Rubina Adrees, Hijaz Ahmad, Sameh Askar

**Affiliations:** 1 Department of Mathematics, University of Management and Technology, Lahore, 54770 Pakistan; 2 Section of Mathematics, International Telematic University Uninettuno, Corso Vittorio Emanuele II, 39, 00186 Rome, Italy; 3 Department of Statistics and Operations Research, College of Science, King Saud University, P.O. Box 2455, Riyadh, 11451 Saudi Arabia

**Keywords:** Engineering, Mathematics and computing, Physics

## Abstract

This article examines the effects of magnetohydrodynamics and heat absorption on an incompressible Jeffrey fluid’ time-dependent free convection flow over an infinite, vertically heated plate with homogeneous heat flux. The constitutive equation for heat flow utilizes the Prabhakar-like fractional derivative. The Laplace transform technique obtains the precise solution for the momentum and thermal profiles. The typical case and well-known outcomes from the literature are retrieved as restraining cases. The graphical analysis of the impact of the flow and fractionalized parameters on the thermal and momentum profiles is presented. Additionally, a comparison is made between the ordinary model and the Prabhakar-like fractional model, which shows that the latter better captures the retention of the physical features of the problem. It is concluded that the Prabhakar-like fractional model is better suited for describing the memory effect of the thermal and momentum fields.

## Introduction

It is common knowledge that various researchers and scientists are keen to study an unsteady viscous fluid’s natural convection due to its various applications, significant properties, and modern technologies. Natural convection properties are illustrated in a wide range of manufacturing sectors because they play a significant contribution in manufacturing; for instance, in nuclear reactors, building ventilation, clay coatings, polymer processing, geothermal systems, waste liquids, food processing industries, greases, nuclear reactors, dehydration, and other emulsions that continue to capture their interest. Several researchers have investigated the volatility of convective flow through a moving vertical plate under variable boundaries^1,2^. Mansour^3^ analyzed the radiative influence of the fluid flow past a sheet. The consequences of natural convective movement on a vertically extended plate under continuous suction were examined by Soundalgekar^4^. Ishak^5,6^ examines how a boundary layer's nonlinear thermal flow affects a horizontal plate. Raptis and Singh^7^ go on to study the freely convective transferring of fluid through an hastening surface. Singh and Kumar^8^ investigated the effect of induced heat transfer on flow in a vertical plate at such a high velocity, taking into account the effect of the magnetic field. Cheng^9^ provides a precise solution to a heat exchange problem involving natural convective from a surface in a flow-permeable medium. Kumari and Pop^10^ define free convection's boundary layer as a homogenous heat flow in a porous medium.^11–15^ and the references therein provide some more recent work on this study.

The word magnetohydrodynamic (MHD) is contained in the word magneto-meaning magnetic, hydro-means water and liquid and dynamic raising to the movement. MHD influences selenology development by influencing magnetic flux appearance, burning, and magnetic field generation concentration. This is important in chemical engineering, meteorology, electronics, metallurgy and meteorology^16^. Seth et al.^17^ examined the significance of chemical reactivity with a radiative heat source on magnetohydrodynamic flow over a sponge medium with time exponential acceleration. Rehman et al.^18^ explored the MHD micropolar liquid flowing over the curve surface. Fetecau et al.^19^ studied the MHD convective flow in a porous material across an infinitely vertically oscillating plate with constant heat flux in incompressible viscous fluids. Many further evaluations of magnetohydrodynamic mixed convective flow under various physical constraints were established. The recent correct solutions for such flows by Vieru^20^, Toki^21^ and Rajesh^22^ are only a few examples. The restricted solutions of MHD via a horizontal oscillating surface with heat flow in a permeable material were found by Samiulhaq et al.^23^ using integral transforms.

Non-Newtonian fluids have recently gained popularity in research because standard Newtonian fluids cannot adequately explain the flow features of various fluids used in engineering and industrial tenders^24,25^. The blood circulation model has effectively employed Jeffrey fluid models because of its viscoelastic behavior, which plays a significant and useful role in biological and fluid mechanics. Newtonian and Maxwell fluids are connected to Jeffrey’s fluid as an exception. A curved elastic surface that produced the MHD of Jeffrey nanomaterial fluid was inspected by Saif et al.^26^ to see if it would move. Khan et al.^27^ examined the time dependence of the natural convection flow of a Jeffrey-type fluid over a long oscillating wall, along with the effects of velocity and temperature. Yasmeen et al.^28^ investigated a quantitative and qualitative method to examine the Jeffrey fluid’s peristaltic flow and Hartmann boundary condition. Sunitha^29^ investigated the double diffusion of a Jeffrey fluid containing gold nano-particles via an asymmetric tube. Dalir^30^ numerically studied the convection flow of a Jeffrey fluid with entropy generation across a stretched sheet. Nadeem et al.^31^ studied stagnation point flow for unsteady oscillations in the Jeffrey fluid. Aziz et al.^32^ discussed the effect of velocity on the unstable MHD mixed convective motion of Jeffrey-based fluid through an indefinitely ramped, closed vertical wall. The flow of Jeffrey material toward a nonlinear stretching surface with variable surface thickness is studied by Hayat et al.^33^ regarding its magnetohydrodynamic at a stagnation point.^34–36^ examined the effects of nanoparticles on the nonlinear Jeffery stream problem using Jeffrey liquids' responses to changing viscosity in an MHD.

Fractional calculus (FC), one of the developments of classical calculus, was given by Leibniz in 1695. In recent years, the FC theory has played an important role in fluid mechanics, biology, mechatronics, electrochemistry, physics, engineering, entropy, rheology and mechanics. Fractional calculus can describe particular engineering processes and physical models more clearly and effectively. Fractional derivatives are Grünwald-Letnikov derivatives, Caputo derivatives, Atangana-Baleanu derivatives, Hadamard derivatives, Caputo-Fabrizio derivatives, Leibnitz derivatives, Liouville and Riemann–Liouville derivatives, Reisz derivatives in various mathematical operators^37,38^. Mittag–Leffler has three parametric functions: the properties of the Prabhakar function discussed by Gara and Garrappa^39^ and investigated thermal transport using Prabhakar memory. Giusti and Colombaro^40^ studied a linear visco-elastic model using the Prabhakar fractional operator. Rehman et al.^41^ studied the generalized Mittag–Leffler function from the exact solution of a free convective flow of Prabhakar fractional Maxwell fluid under Newtonian heating. The majority of works^42,43^, and^44^ are concerned with flow issues involving diverse fluids, fractional operators and heat transport occurrences.

The present work investigates a Prabhakar-like fractional Jeffrey-type fluid that moves across an infinite vertical surface with uniform heat flux. The purpose of this novel mathematical fractional model is to consider generalized effects and propose the Prabhakar method with generalizations of Fourier’s law. The constitutive equations employed the Prabhakar derivative to account for the generalized memory effects. In the end, an appraisal between the fractional Jeffrey fluid, fractional viscous fluid, and ordinary Jeffrey fluid, ordinary viscous fluid is also given. It can be seen that fractional Jeffrey fluid and fractional viscous fluid move more slowly than ordinary Jeffrey fluid and ordinary viscous fluid. Further, the physical effects of various fractional and fluid parameters have been analyzed graphically by Mathcad software. Prabhakar operators with coefficients may be beneficial in choosing a mathematical model that can account for both experimental and hypothetical data.

## Mathematical model

**Figure 1:**
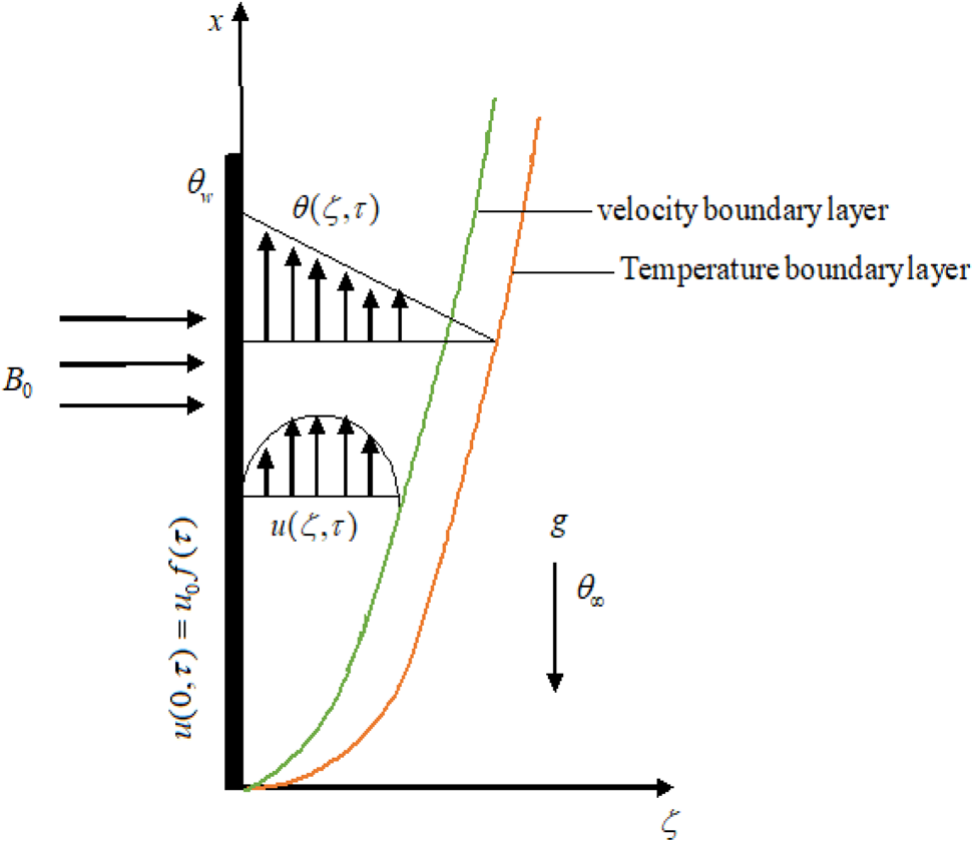
Flow geometry.

The analysis involves studying the unsteady, incompressible, electrically conducting free convective flow of a Jaffrey fluid over an endless vertical plate, where the plate is oriented vertically along the x-axis, and the y-axis is perpendicular to it as shown in Fig. 1. The system starts at a constant temperature $\theta_{\infty }$ with the fluid and plate resting. For a time, $\tau = 0^{ + } ,$ the plate starts to move in its plane $\left( {\zeta = 0} \right)$ accordingly, $\vec{u} = u_{0} f\left( \tau \right)i,\,\,\tau > 0,$ where $u_{0}$ denotes the constant velocity, $i$ denotes the unit vector in the flow direction. Since the shear caused the fluid to flow slowly, it obtained fluid velocity in the form ${\mathbf{U}}(\zeta ,\tau ) = u(\zeta ,\tau )i.$

Assuming the conventional Boussineq's approximation and the conditions mentioned above, the governing equations for the fluid velocity and thermal transport are given by^27,45,46^.

- Momentum Equation
  1 $$\rho \frac{\partial u(\zeta ,\tau )}{{\partial \tau }} = \frac{\mu }{{1 + \lambda_{1} }}\left[ {1 + \lambda \frac{\partial }{\partial \tau }} \right]\frac{{\partial^{2} u(\zeta ,\tau )}}{{\partial \zeta^{2} }} + g\rho \beta_{1} \left[ {\theta (\zeta ,\tau ) - \theta_{\infty } } \right] - \sigma B_{o}^{2} u(\zeta ,\tau ),\,\,\,\,\,\zeta ,\,\,\tau > 0,$$
- Energy balance equation
  2 $$\rho c_{p} \frac{\partial \theta (\zeta ,\tau )}{{\partial \tau }} = - \frac{\partial q(\zeta ,\tau )}{{\partial \zeta }} - Q\left[ {\theta (\zeta ,\tau ) - \theta_{\infty } } \right],\,\,\,\,\zeta ,\,\,\tau > 0,$$
- Fourier’s Law of thermal flux
  3 $$q\left( {\zeta ,\tau } \right) = - k\frac{{\partial \theta \left( {\zeta ,\tau } \right)}}{\partial \zeta },\,\,\,\,\zeta ,\,\,\,\tau > 0,$$

The relevant initial/ boundary condition

4 $$u\left( {\zeta ,0} \right) = 0,\,\,\,\theta \left( {\zeta ,0} \right) = \theta_{\infty } ,\,\,\,\zeta \ge 0,$$

5 $$u(0,\tau ) = u_{0} f(\tau ),\,\,\,\,\left. {\frac{\partial \theta (\zeta ,\tau )}{{\partial \zeta }}} \right|_{\zeta = 0} = - \frac{{q_{0} }}{k},\,\,\tau > 0,$$

6 $$u\left( {\zeta ,\tau } \right) \to 0,\,\,\,\theta \left( {\zeta ,\tau } \right) \to \theta_{\infty } ,\,\,\,\zeta \to \infty ,\,\,\,\tau > 0.$$

Presenting the dimensionless parameters as follows:

$$u^{ * } = \frac{u}{{u_{0} }},\,\,\zeta^{ * } = \frac{{u_{0} \zeta }}{\nu },\,\,\tau^{ * } = \frac{{u_{0}^{2} \tau }}{\nu },\,\,\theta^{ * } = \frac{{u_{0} k}}{{\nu q_{0} }}(\theta - \theta_{\infty } ),\,\,f(\tau^{ * } ) = f\left( {\frac{{u_{0}^{2} \tau }}{\nu }} \right),\,\,q^{ * } = \frac{q}{{q_{0} }},\,\,Gr = \left( {\frac{\nu }{{u_{0}^{2} }}} \right)^{2} \frac{{g\beta_{1} q_{0} }}{k},$$

7 $$\Pr = \frac{{\mu c_{p} }}{k},\,\,M = \frac{{B_{0} \sigma \nu^{2} }}{{\mu u_{0}^{2} }},\,\,\lambda^{ * } = \frac{{\lambda u_{0}^{2} }}{\nu },\,\,Q_{0}^{ * } = \frac{\nu Q}{{ku_{0}^{2} }}.$$

By substituting into Eqs. (1)–(6), the subsequent dimensionless governing equations are obtained, and for easiness, the asterisk notation “*” is avoided:

8 $$\frac{\partial u(\zeta ,\tau )}{{\partial \tau }} = \frac{1}{{1 + \lambda_{1} }}\left[ {1 + \lambda \frac{\partial }{\partial \tau }} \right]\frac{{\partial^{2} u(\zeta ,\tau )}}{{\partial \zeta^{2} }} + Gr\theta (\zeta ,\tau ) - Mv(\zeta ,\tau ),$$

9 $$\Pr \frac{\partial \theta (\zeta ,\tau )}{{\partial \tau }} = - \frac{\partial q(\zeta ,\tau )}{{\partial \zeta }} - Q_{o} \theta (\zeta ,\tau ),$$

10 $$q(\zeta ,\tau ) = - \frac{\partial \theta (\zeta ,\tau )}{{\partial \zeta }},$$

with the non-dimensional conditions

11 $$u(\zeta ,0) = 0,\,\,\theta (\zeta ,0) = 0,\,\,\,\,\zeta \ge 0,$$

12 $$u(0,\tau ) \to f\left( \tau \right),\,\,\left. {\frac{\partial \theta (\zeta ,\tau )}{{\partial \zeta }}} \right|_{\zeta = 0} = - 1,\,\,\,\tau > 0,$$

13 $$u(\zeta ,\tau ) \to 0,\,\,\theta (\zeta ,\tau ) \to 0\,\,\,{\text{as}}\,\,\,\zeta \to \infty ,\,\,\,\,\,\,\tau > 0.$$

In this study, the momentum and energy equations were solved using a reliable fractional mathematical model known as the regularized Prabhakar fractional derivative, which can be mathematically expressed as^39,40^:

Let

$$0 < b < m,\,\,$$ where $$m \in Z$$ and $$h \in AC^{q} (o,b)$$

14 $$^{C} D_{\alpha ,\,\,\beta ,\,\,a}^{\gamma } h(t) = E_{\alpha ,\,\,m - \beta ,\,\,a}^{ - \gamma } h^{(q)} (t) = e_{\alpha ,\,\,q - \beta }^{ - \gamma } (a;t) * h^{(q)} (t) = \int\limits_{0}^{t} {(t - \zeta )^{q - \beta - 1} } E_{\alpha ,\,\,q - \beta }^{ - \gamma } (a(t - \zeta )^{\alpha } )h^{(q)} (\zeta )d\zeta ,$$

where

$$E_{\alpha ,\,\,\beta ,\,\,a}^{\gamma } h(t) = \int\limits_{0}^{t} {(t - \zeta )^{\beta - 1} E_{\alpha ,\,\beta }^{\gamma } (a(t - \zeta )^{\alpha } )h(\zeta )d\zeta } ,$$

Characterized the Prabhakar integral, and

$$E_{\alpha ,\,\,\beta }^{\gamma } (z) = \sum\limits_{n = 0}^{\infty } {\frac{{\Gamma \left( {\gamma + n} \right)z^{n} }}{{n!\Gamma \left( \gamma \right)\Gamma \left( {\alpha n + \beta } \right)}},\,\,\,\alpha ,\,\,\beta ,\,\,\gamma ,\,\,z \in } \,C,\,\,{\text{Re}} \left( \alpha \right) > 0,$$

is the three-parametric Mittag–Leffler function Moreover, the function $e_{\alpha ,\,\,\beta }^{\gamma } (a;\,\,t) = t^{\beta - 1} E_{\alpha ,\,\,\beta }^{\gamma } \left( {at^{a} } \right)$ with $\,t \in \Re ,\,\,\,\alpha ,\,\,\,\beta ,\,\,\,\gamma ,\,\,\,a \in C,\,\,{\text{Re}} \left( \alpha \right) > 0,$ is known as the Prabhakar kernel.

The Laplace transformation of the regularized Prabhakar fractional derivative operator $^{C} D_{\alpha ,\,\,\beta ,\,\,a}^{\gamma } \left( \cdot \right)$ is given by

15 $$\begin{aligned} L\left\{ {^{C} D_{\alpha ,\beta ,a}^{\gamma } h(t)} \right\} & = L\left\{ {e_{\alpha ,q - \beta }^{ - \gamma } (a;t) * h^{(q)} (t)} \right\} = L\left\{ {e_{\alpha ,q - \beta }^{ - \gamma } (a;t)} \right\}L\left\{ {\,h^{(q)} (t)} \right\} \\ & = s^{\beta - q} (1 - as^{ - \alpha } )^{\gamma } L\left\{ {h^{(q)} (t)} \right\}. \\ \end{aligned}$$

If we set $\beta = \gamma = 0,$ we can recover the classical Fourier’s law. As the Prabhakar fractional derivative relies on Fourier’s law of heat transfer, Fourier's law can be expressed using the regularized Prabhakar fractional derivative as follows:

16 $$q(\zeta ,\tau ) = -^{C} D_{\alpha ,\beta ,a}^{\gamma } \frac{\partial \theta (\zeta ,\tau )}{{\partial \zeta }}.$$

## Solutions to the problem

### Thermal transport field

Applying the Laplace transform to Eqs. (9), (12)_2_, (13)_2,_ and (16) and using condition (11)_2_, we attain the transmuted problem:

17 $$(\Pr s + Q_{0} )\tilde{\theta }(\zeta ,s) = - \frac{{\partial \tilde{q}(\zeta ,s)}}{\partial \zeta },$$

18 $$\tilde{q}\left( {\zeta ,s} \right) = - s^{\beta } \left( {1 - as^{ - \alpha } } \right)^{\gamma } \tilde{\theta }\left( {\zeta ,s} \right),$$

19 $$\frac{{\partial \tilde{\theta }(0,s)}}{\partial \zeta } = - \frac{1}{s},\tilde{\theta }(\zeta ,s) \to 0,\zeta \to \infty ,$$

where $\,\tilde{\varphi }(\eta ,s) = \int_{0}^{\infty } {\varphi \left( {\eta ,t} \right)e^{ - st} dt}$ specify the Laplace transform of the function $\varphi (\eta ,t),$ with alter parameter $s$.

Using Eq. (18) in Eq. (17), we get

20 $$\,\frac{{\partial^{2} \tilde{\theta }(\zeta ,s)}}{{\partial \zeta^{2} }} - \frac{{\Pr s + Q_{0} }}{{s^{\beta } (1 - as^{ - \alpha } )^{\gamma } }}\tilde{\theta }(\zeta ,s) = 0.$$

Solving differential Eq. (20) subject to boundary conditions (19) yields the following solution:

21 $$\widetilde{\theta }(\zeta ,s) = \frac{1}{{s\sqrt {A_{0} \left( s \right)} }}e^{{ - \zeta \sqrt {A_{0} \left( s \right)} }} ,$$

where $$A_{0} \left( s \right) = \frac{{\Pr s + Q_{0} }}{{s^{\beta } (1 - as^{ - \alpha } )^{\gamma } }}.$$

After using the exponential function series formula, $e^{x} = \sum\nolimits_{k = 0}^{\infty } {\frac{{x^{k} }}{k!}} ,$ Eq. (21) takes the following equivalent form:

22 $$\widetilde{\theta }(\zeta ,s) = \sum\limits_{k = 0}^{\infty } {\frac{{( - \zeta )^{k} \Pr^{{\frac{ - 1 + k}{2}}} }}{k!}} s^{{ - \left( {1 + \frac{{^{\beta ( - 1 + k)} }}{2}} \right)}} (1 - as^{ - \alpha } )^{{\frac{ - \gamma (k - 1)}{2}}} s^{{\frac{k - 1}{2}}} \left( {1 + \frac{{Q_{0} }}{\Pr }s^{ - 1} } \right)^{{\frac{ - 1 + k}{2}}} .$$

By applying the inverse Laplace transform to Eq. (22), the solution for the thermal field is obtained as follows:

23 $$\theta (\zeta ,\tau ) = \sum\limits_{k = 0}^{\infty } {\frac{{( - \zeta )^{k} \Pr^{{\frac{k - 1}{2}}} }}{k!}} e_{{\alpha ,\frac{{^{\beta (k - 1)} }}{2} + 1}}^{{\frac{\gamma (k - 1)}{2}}} (a;\tau ) * e_{{1,\frac{1 - k}{2}}}^{{\frac{1 - k}{2}}} \left( {\frac{{Q_{0} }}{\Pr };\tau } \right).$$

### Thermal field for the classical case $\left( {\beta = \gamma = 0} \right)$

In this distinct case, when $\beta = \gamma = 0,$ we acquire the following classical thermal field;

25 $$\theta (\zeta ,\tau ) = \sum\limits_{k = 0}^{\infty } {\frac{{\left( { - \zeta } \right)^{k} \Pr^{{\frac{k - 1}{2}}} }}{k!}e_{{1,\frac{3 - k}{2}}}^{{\frac{1 - k}{2}}} \left( {\frac{{Q_{0} }}{\Pr };\tau } \right)} = \sum\limits_{k = 0}^{\infty } {\frac{{\left( { - \zeta } \right)^{k} \Pr^{{\frac{k - 1}{2}}} }}{k!}\tau^{{\frac{1 - k}{2}}} E_{{1,\frac{3 - k}{2}}}^{{\frac{1 - k}{2}}} \left( {\frac{{Q_{0} }}{\Pr }\tau } \right)} .$$

Taking $Q_{0} = 0,$ into Eqs. (23) and (25), we obtain results similar to those conquered by Sun et al.^47^, Eqs. (23) and (26).

### Velocity field

Applying the Laplace transform to Eqs. (8), (12)_1_, (13)_1,_ and (21) and using condition (11)_1_, we acquire the resulting transmuted problem:

26 $$\frac{{\partial^{2} \tilde{u}(\zeta ,s)}}{{\partial \zeta^{2} }} - (M + s)\frac{{1 + \lambda_{1} }}{1 + \lambda s}\tilde{u}(\zeta ,s) = - Gr\frac{1}{{s\sqrt {A_{0} \left( s \right)} }}\left( {\frac{{1 + \lambda_{1} }}{1 + \lambda s}} \right)e^{{ - \zeta \sqrt {A_{0} \left( s \right)} }} ,$$

27 $$\tilde{u}(0,s) = \tilde{f}(s),\,\,\,\,\tilde{u}(\zeta ,s) \to 0,\,\,\,\,\zeta \to \infty .$$

Solving differential Eq. (26) subject to boundary conditions (27) yields the following solution:

28 $$\begin{aligned} \tilde{u}(y,s) & = \tilde{f}\left( s \right) \cdot \exp \left( { - \zeta \sqrt {\left( {M + s} \right)\left( {\frac{{1 + \lambda_{1} }}{1 + \lambda s}} \right)} } \right) + \frac{{Gr\left( {\frac{{1 + \lambda_{1} }}{1 + \lambda s}} \right)}}{{\sqrt {A_{0} (s)} \left( {\left( {M + s} \right)\left( {\frac{{1 + \lambda_{1} }}{1 + \lambda s}} \right) - A_{0} \left( s \right)} \right)}} \\ & \quad \times \left[ {\frac{1}{s}e^{{ - \zeta \sqrt {A_{0} \left( s \right)} }} - \frac{1}{s}e^{{ - \zeta \sqrt {\left( {M + s} \right)\left( {\frac{{1 + \lambda_{1} }}{1 + \lambda s}} \right)} }} } \right]. \\ \end{aligned}$$

To calculate the inverse Laplace transform, Eq. (28) can be written as:

29 $$\tilde{u}\left( {\zeta ,s} \right) = \tilde{u}_{1} \left( {\zeta ,s} \right) + \tilde{u}_{2} \left( s \right) \cdot \left[ {\tilde{u}_{3} \left( {\zeta ,s} \right) - \tilde{u}_{4} \left( {\zeta ,s} \right)} \right].$$

where

30 $$\begin{aligned} \tilde{u}_{1} (\zeta ,s) & = \tilde{f}(s) \cdot \exp \left( { - \zeta \sqrt {\left( {M + s} \right)\left( {\frac{{1 + \lambda_{1} }}{1 + \lambda s}} \right)} } \right), \\ \tilde{u}_{2} (s) & = \frac{Gr}{{\sqrt {A_{0} \left( s \right)} \left( {1 - A_{0} \left( s \right)\left( {M + s} \right)^{ - 1} \left( {\frac{{1 + \lambda_{1} }}{1 + \lambda s}} \right)^{ - 1} } \right)\left( {M + s} \right)}}, \\ \tilde{u}_{3} (\zeta ,s) & = \frac{1}{s}\exp \left( { - \zeta \sqrt {A_{0} \left( s \right)} } \right),\tilde{u}_{4} (\zeta ,s) = \exp \left( { - \zeta \sqrt {\left( {M + s} \right)\left( {\frac{{1 + \lambda_{1} }}{1 + \lambda s}} \right)} } \right). \\ \end{aligned}$$

The inverse Laplace transform of Eq. (29) can be acquired by utilizing equation (A.1) from the appendix and the convolution theorem, expressed as:

31 $$u\left( {\zeta ,\tau } \right) = u_{1} \left( {\zeta ,\tau } \right) + u_{2} \left( \tau \right) * \left[ {u_{3} \left( {\zeta ,\tau } \right) - u_{4} \left( {\zeta ,\tau } \right)} \right].$$

where “$*$” indicate the convolution product

$$u_{1} (\zeta ,\tau ) = f^{\prime } \left( \tau \right) * \left[ \begin{gathered} \frac{1}{\lambda }e^{{ - \frac{\tau }{\lambda }}} \int\limits_{0}^{\infty } {e^{{ - \frac{u}{\lambda }}} } erfc\left( {\frac{{\zeta \sqrt {1 + \lambda_{1} } }}{2\lambda \sqrt u }} \right)I_{0} \left( {\frac{2}{\lambda }\sqrt {\left( {1 - M\lambda } \right)u\tau } } \right)du \hfill \\ + \frac{M}{\lambda }\int\limits_{0}^{\infty } {\int\limits_{0}^{\tau } {e^{{ - \frac{u + q}{\lambda }}} erfc\left( {\frac{{\zeta \sqrt {1 + \lambda_{1} } }}{2\lambda \sqrt u }} \right)I_{0} \left( {\frac{2}{\lambda }\sqrt {\left( {1 - M\lambda } \right)uq} } \right)dqdu} } \hfill \\ \end{gathered} \right],$$

$$u_{2} (\tau ) = Gr\sum\limits_{k = 0}^{\infty } {\sum\limits_{m = 0}^{\infty } {\left( \begin{gathered} k \hfill \\ m \hfill \\ \end{gathered} \right)} } \frac{{\Pr^{{k - \frac{1}{2}}} }}{{(1 + \lambda_{1} )^{k} }}\lambda^{m} e_{{\alpha ,\beta \left( {\frac{1}{2} - k} \right) - m}}^{{\gamma \left( {\frac{1}{2} - k} \right)}} \left( {a;\tau } \right) * e_{{1,\frac{1}{2} - k}}^{{\frac{1}{2} - k}} \left( {\frac{{Q_{0} }}{\Pr };\tau } \right) * e_{1,k + 1}^{k + 1} \left( {M;\tau } \right),$$

$$u_{3} (\zeta ,\tau ) = \sum\limits_{k = 0}^{\infty } {\frac{{( - \zeta )^{k} \Pr^{\frac{k}{2}} }}{k!}} e_{{\alpha ,\frac{\beta k}{2} + 1}}^{{\frac{ - \gamma k}{2}}} \left( {a;\tau } \right) * e_{{1, - \frac{k}{2}}}^{{ - \frac{k}{2}}} \left( {\frac{{Q_{0} }}{\Pr };\tau } \right),$$

$$\begin{aligned} u_{4} (\zeta ,\tau ) & = \frac{1}{\lambda }e^{{ - \frac{\tau }{\lambda }}} \int\limits_{0}^{\infty } {e^{{ - \frac{u}{\lambda }}} } erfc\left( {\frac{{\zeta \sqrt {1 + \lambda_{1} } }}{2\sqrt u }} \right)I_{0} \left( {\frac{2}{\lambda }\sqrt {\left( {1 - M\lambda } \right)u\tau } } \right)du \\ & \quad + \frac{M}{\lambda }\int\limits_{0}^{\infty } {\int\limits_{0}^{\tau } {e^{{ - \frac{u + q}{\lambda }}} erfc\left( {\frac{{\zeta \sqrt {1 + \lambda_{1} } }}{2\sqrt u }} \right)I_{0} \left( {\frac{2}{\lambda }\sqrt {\left( {1 - M\lambda } \right)uq} } \right)dqdu} } . \\ \end{aligned}$$

#### Velocity field for ordinary Jeffery fluid $\left( {\beta = \gamma = 0} \right)$

In the specific scenario where $\beta = \gamma = 0,$ the velocity profile of the ordinary Jeffrey fluid, as given in Eq. (28), can be expressed as follows:

32 $$\begin{aligned} \tilde{u}(\zeta ,s) & = \tilde{f}\left( s \right) \cdot \exp \left( { - \zeta \sqrt {\left( {M + s} \right)\left( {\frac{{1 + \lambda_{1} }}{1 + \lambda s}} \right)} } \right) + \frac{{Gr\left( {\frac{{1 + \lambda_{1} }}{1 + \lambda s}} \right)}}{{\sqrt {\left( {\Pr s + Q_{0} } \right)} \left( {\left( {M + s} \right)\left( {\frac{{1 + \lambda_{1} }}{1 + \lambda s}} \right) - \left( {\Pr s + Q_{0} } \right)} \right)}} \\ & \quad \times \left[ {\frac{1}{s}e^{{ - \zeta \sqrt {\left( {\Pr s + Q_{0} } \right)} }} - \frac{1}{s}e^{{ - \zeta \sqrt {\left( {M + s} \right)\left( {\frac{{1 + \lambda_{1} }}{1 + \lambda s}} \right)} }} } \right]. \\ \end{aligned}$$

To calculate the inverse Laplace transform of Eq. (32), equation (A.2) from the appendix can be utilized, followed by the application of the convolution theorem:

33 $$u\left( {\zeta ,\tau } \right) = u_{11} \left( {\zeta ,\tau } \right) + u_{12} \left( \tau \right) * \left[ {u_{13} \left( {\zeta ,\tau } \right) - u_{14} \left( {\zeta ,\tau } \right)} \right],$$

where

$$u_{11} \left( {\zeta ,\tau } \right) = u_{1} \left( {\zeta ,\tau } \right),\;\left( {{\text{Eq}}. \left( {{31}} \right)} \right)$$

$$u_{12} (\tau ) = Gr\sum\limits_{k = 0}^{\infty } {\sum\limits_{m = 0}^{\infty } {\left( \begin{gathered} k \hfill \\ m \hfill \\ \end{gathered} \right)} } \Pr^{{k - \frac{1}{2}}} b_{0} \lambda^{m} e_{{1,\left( {\frac{1}{2} - k} \right)}}^{{\left( {\frac{1}{2} - k} \right)}} \left( {\frac{{Q_{0} }}{\Pr };\tau } \right) * e_{1,k + 1}^{k + 1} \left( {M;\tau } \right) * \frac{1}{{\tau^{m} \Gamma \left( {1 - m} \right)}},$$

$$u_{13} (\zeta ,\tau ) = \frac{1}{2}\left[ {e^{{ - \zeta \sqrt {Q_{0} } }} erfc\left( {\frac{{\zeta \sqrt {\Pr } }}{2\sqrt \tau } - \sqrt {\frac{{Q_{0} }}{\Pr }\tau } } \right) + e^{{\zeta \sqrt {Q_{0} } }} erfc\left( {\frac{{\zeta \sqrt {\Pr } }}{2\sqrt \tau } + \sqrt {\frac{{Q_{0} }}{\Pr }\tau } } \right)} \right],\,$$

$$u_{14} (\zeta ,\tau ) = u_{4} (\zeta ,\tau ),\;({\text{Eq}}. \left( {{31}} \right)).$$

#### Velocity field for fractional viscous fluid $\left( {\lambda_{1} ,\,\,\lambda \to 0} \right)$

If $\lambda_{1} ,\,\,\lambda \to 0,$ the velocity profile of the fractional viscous fluid can be obtained from Eq. (28) as follows:

34 $$\tilde{u}(\zeta ,s) = \tilde{f}\left( s \right) \cdot \exp \left( { - \zeta \sqrt {\left( {M + s} \right)} } \right) + \frac{Gr}{{\sqrt {A_{0} (s)} \left( {\left( {M + s} \right) - A_{0} \left( s \right)} \right)}}\left[ {\frac{1}{s}e^{{ - \zeta \sqrt {A_{0} \left( s \right)} }} - \frac{1}{s}e^{{ - \zeta \sqrt {\left( {M + s} \right)} }} } \right].$$

By employing equation (A.2) from the appendix to calculate the inverse Laplace transform of Eq. (34), followed by the application of the convolution theorem:

35 $$u\left( {\zeta ,\tau } \right) = u_{21} \left( {\zeta ,\tau } \right) + u_{22} \left( \tau \right) * \left[ {u_{23} \left( {\zeta ,\tau } \right) - u_{24} \left( {\zeta ,\tau } \right)} \right],$$

where

$$\begin{gathered} u_{21} (\zeta ,\tau ) = f^{\prime}(\tau ) * \frac{1}{2}\left[ {e^{\zeta \sqrt M } erfc\left( {\frac{\zeta }{2\sqrt \tau } + \sqrt {M\tau } } \right) + e^{ - \zeta \sqrt M } erfc\left( {\frac{y}{2\sqrt \tau } - \sqrt {M\tau } } \right)} \right], \hfill \\ \hfill \\ \end{gathered}$$

$$u_{22} (\tau ) = \sum\limits_{k = 0}^{\infty } {\Pr^{{k - \frac{1}{2}}} } e_{\alpha ,\beta k}^{\gamma k} \left( {a;\tau } \right) * e_{{1,\frac{1}{2} - k}}^{{\frac{1}{2} - k}} \left( {\frac{{Q_{0} }}{\Pr };\tau } \right) * e_{1,k + 1}^{k + 1} \left( {M;\tau } \right),$$

$$u_{23} (\zeta ,\tau ) = \sum\limits_{k = 0}^{\infty } {\frac{{( - \zeta )^{k} \Pr^{\frac{k}{2}} }}{k!}} e_{{\alpha ,\frac{\beta k}{2} + 1}}^{{\frac{ - \gamma k}{2}}} \left( {a;\tau } \right) * e_{{1, - \frac{k}{2}}}^{{ - \frac{k}{2}}} \left( {\frac{{Q_{0} }}{\Pr };\tau } \right),$$

$$u_{24} (\zeta ,\tau ) = \frac{1}{2}\left[ {e^{\zeta \sqrt M } erfc\left( {\frac{\zeta }{2\sqrt \tau } + \sqrt {M\tau } } \right) + e^{ - \zeta \sqrt M } erfc\left( {\frac{y}{2\sqrt \tau } - \sqrt {M\tau } } \right)} \right].$$

#### Velocity field for classical viscous fluid $\left( {\lambda_{1} ,\,\,\lambda \to 0,\,\,\beta = \gamma = 0} \right)$

When $\lambda ,\lambda_{1} \to 0$ and $\beta = \gamma = 0,$ the classical viscous fluid can be obtained from Eq. (28) as follows:

36 $$\begin{aligned} \tilde{u}(\zeta ,s) & = \tilde{f}\left( s \right) \cdot \exp \left( { - \zeta \sqrt {\left( {M + s} \right)} } \right) \\ & \quad + \frac{Gr}{{\sqrt {\Pr s + Q_{0} } \left( {\left( {M + s} \right) - \left( {\Pr s + Q_{0} } \right)} \right)}}\left[ {\frac{1}{s}e^{{ - \zeta \sqrt {\Pr s + Q_{0} } }} - \frac{1}{s}e^{{ - \zeta \sqrt {\left( {M + s} \right)} }} } \right]. \\ \end{aligned}$$

The inverse Laplace transform of Eq. (36) is;

37 $$u\left( {\zeta ,\tau } \right) = u_{31} \left( {\zeta ,\tau } \right) + u_{32} \left( \tau \right) * \left[ {u_{33} \left( {\zeta ,\tau } \right) - u_{34} \left( {\zeta ,\tau } \right)} \right],$$

where

$$u_{31} (\zeta ,\tau ) = u_{21} (\zeta ,\tau ),$$

$$u_{32} (\tau ) = \sum\limits_{k = 0}^{\infty } {\Pr^{{k - \frac{1}{2}}} } e_{{1,\frac{1}{2} - k}}^{{\frac{1}{2} - k}} \left( {\frac{{Q_{0} }}{\Pr };\tau } \right) * e_{1,k + 1}^{k + 1} \left( {M;\tau } \right),$$

$$u_{33} (\zeta ,\tau ) = u_{13} (\zeta ,\tau ),$$

$$u_{42} (\zeta ,\tau ) = u_{24} (\zeta ,\tau ).$$

When dealing with complex transform fields, obtaining the inverse transform sequentially for practical or physical applications is not always feasible. Therefore, several numerical methods, such as the inverse Laplace transform, have been employed to obtain the required result. Some recent studies^1,27,48–50^ have utilized the Gaver–Stehfest algorithm methods^51,52^ to solve fractional differential equations as an effective approach for numerical Laplace methods. The Gaver-Stehfest algorithm^52^ can be expressed mathematically as follows:

38 $$\begin{aligned} \theta_{s} (\varphi ,\tau ) & = \frac{\ln (2)}{\tau }\sum\limits_{i = 1}^{2n} {d_{i} } \widetilde{\theta }\left( {\varphi ,i\frac{\ln (2)}{\tau }} \right), \\ u_{s} \left( {\zeta ,\tau } \right) & = \frac{\ln (2)}{\tau }\sum\limits_{l = 1}^{2n} {d_{i} } \tilde{u}\left( {q,i\frac{\ln (2)}{\tau }} \right), \\ \end{aligned}$$

where

$$d_{i} = \left( { - 1} \right)^{i + n} \sum\limits_{{p = \left[ {\frac{j + 1}{2}} \right]}}^{\min (j,n)} {\frac{{p^{n} (2n)!}}{(n - p)!k!(p - 1)!(i - p)!(2p - i)!}} ,$$

The bracket function $\left[ p \right]$ represents the integer value function and is a positive integer $n$. To verify the accuracy of our numerical results and compare them to those obtained using the Stehfest technique, we used Tzou’s approach^53^ as an alternative approximation for the solution of the temperature and velocity fields. Mathematically, Tzou's technique can be stated as follows.

$$\begin{aligned} \theta_{T} (\varphi ,\tau ) & = \frac{{e^{4.7} }}{\tau }\left[ {\frac{1}{2}\widetilde{\theta }\left( {\varphi ,\frac{4.7}{\tau }} \right)} \right] + {\text{Re}} \sum\limits_{l = 1}^{N} {\left( { - 1} \right)^{l} } \widetilde{\theta }\left( {\varphi ,\frac{4.7 + l\pi i}{\tau }} \right), \\ u_{T} \left( {\zeta ,\tau } \right) & = \frac{{e^{4.7} }}{\tau }\left[ {\frac{1}{2}\tilde{v}\left( {\varphi ,\frac{4.7}{\tau }} \right)} \right] + {\text{Re}} \sum\limits_{l = 1}^{N} {\left( { - 1} \right)^{l} } \tilde{u}\left( {\varphi ,\frac{4.7 + l\pi i}{\tau }} \right), \\ \end{aligned}$$

where ${\text{Re}} \left( \cdot \right)$ is the real part, and $N$ is the natural number, $i$ is the imaginary unit.

## Result and discussion

**Figure 2:**
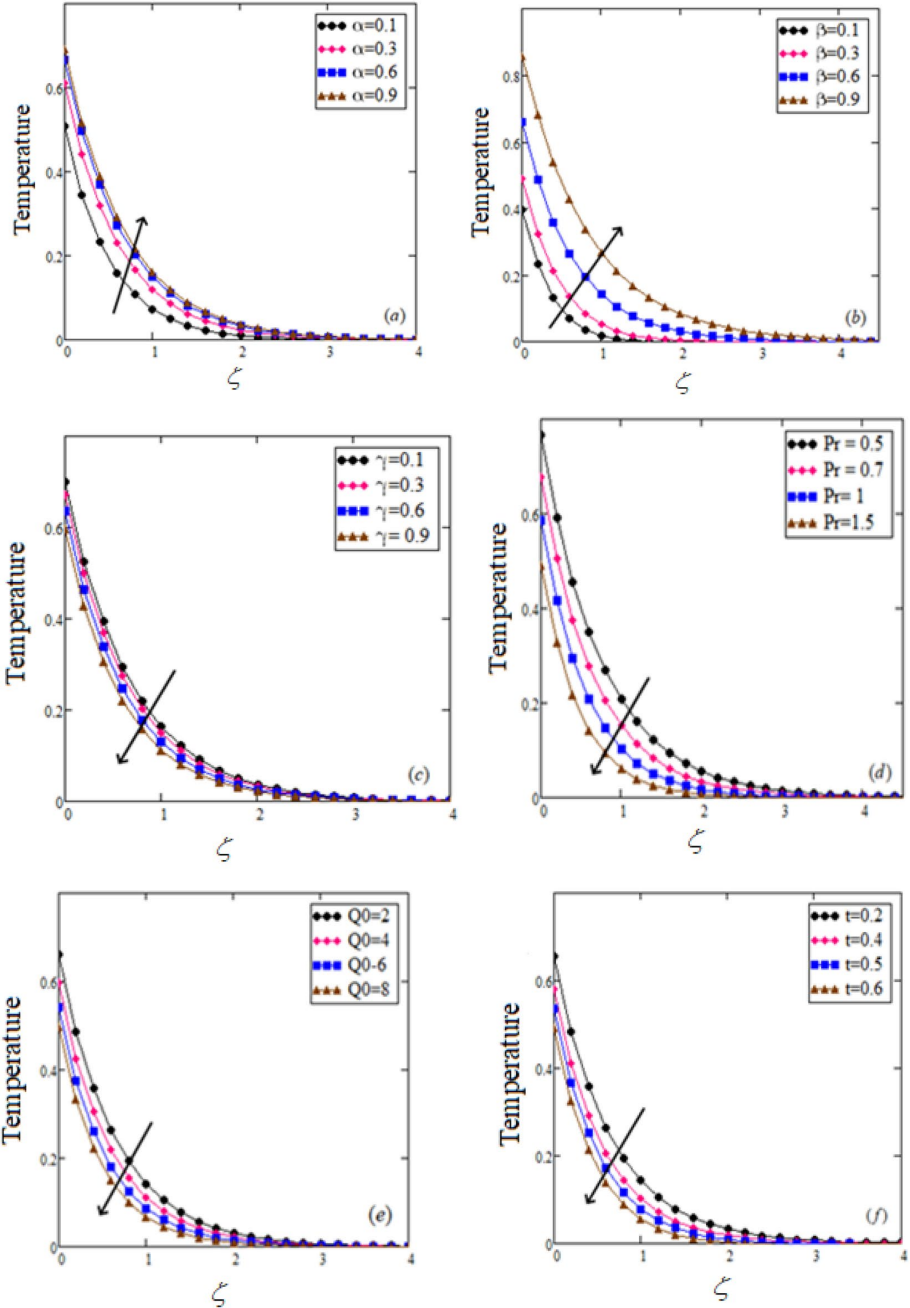
Temperature profile $\theta (\zeta ,t)$ for Jeffery fluid at $Q_{0} = 2,\,\,\Pr = 0.75,\,\,\alpha = 0.5,\,\,\gamma = 0.4\,,\,\,\beta = 0.6.$

**Figure 3:**
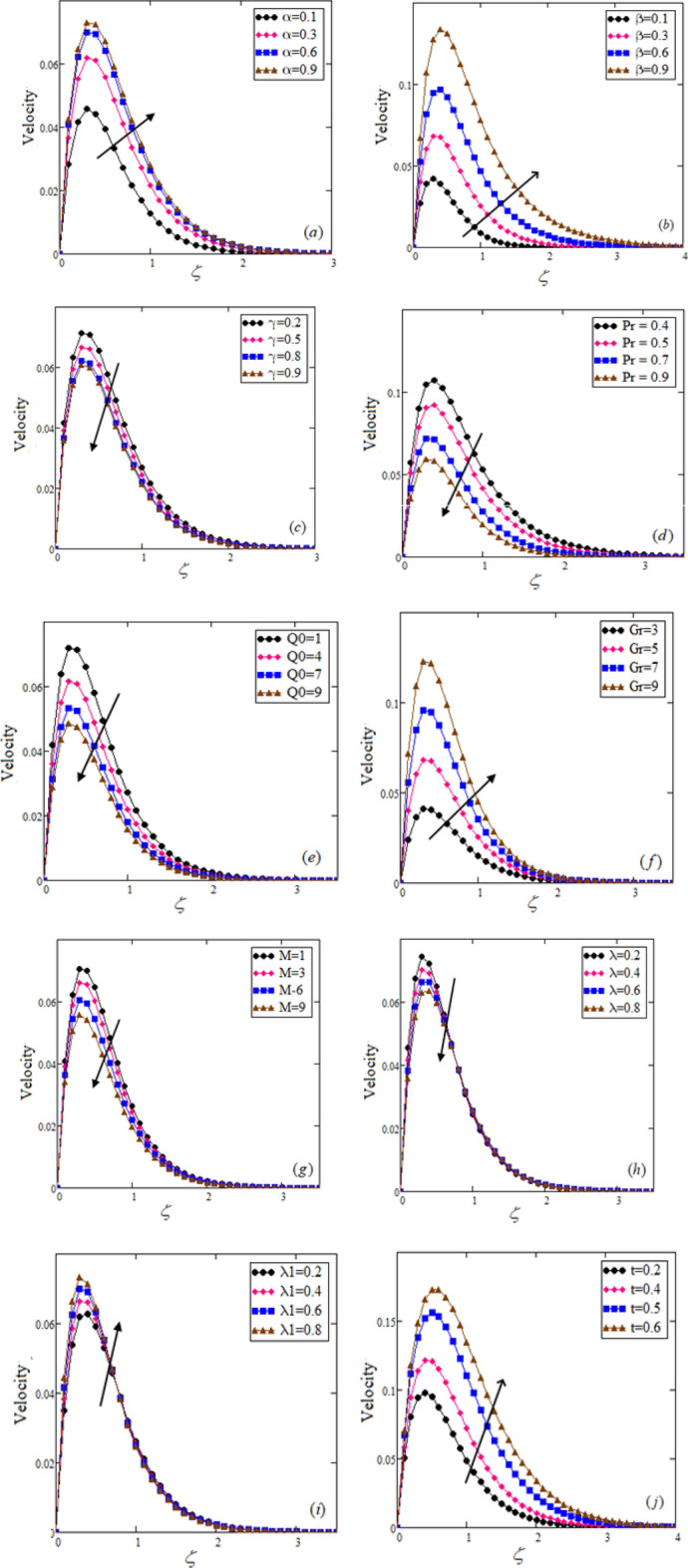
Velocity profile $u(\zeta ,\tau )$ for Jeffery fluid at $\alpha = 0.5,\,\,\beta = 0.6\,,\,\,\gamma = 0.4,\,\,\Pr = 0.75,\,\,Q_{0} = 2,\,\,M = 2,$ $Gr = 5,\,\,\lambda = 0.5,\,\,\lambda_{1} = 0.5,\,\,t = 0.1.$

**Figure 4:**
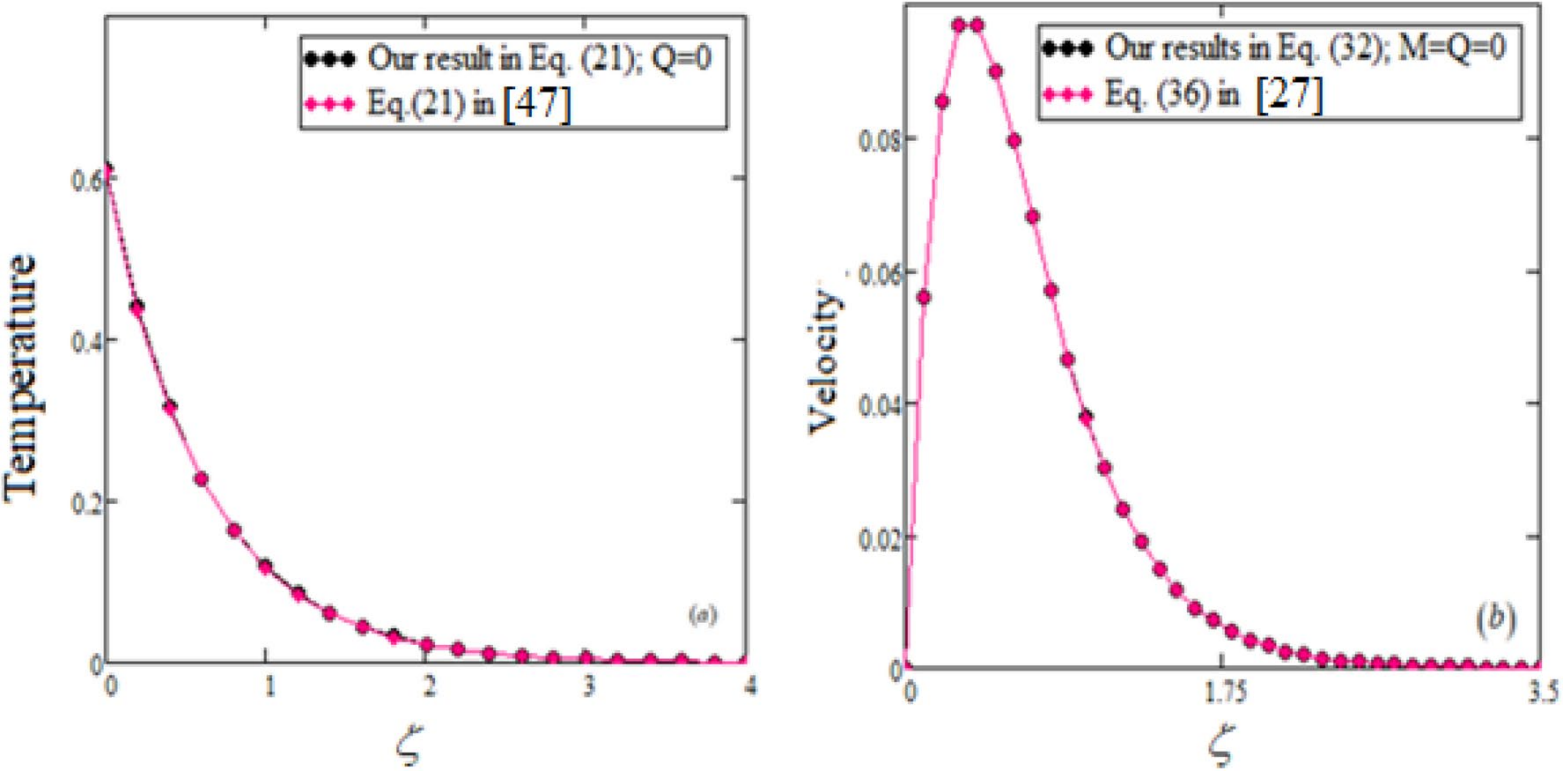
Comparison of our result with (Eqs. (21) and (36),^27,47^).

**Figure 5:**
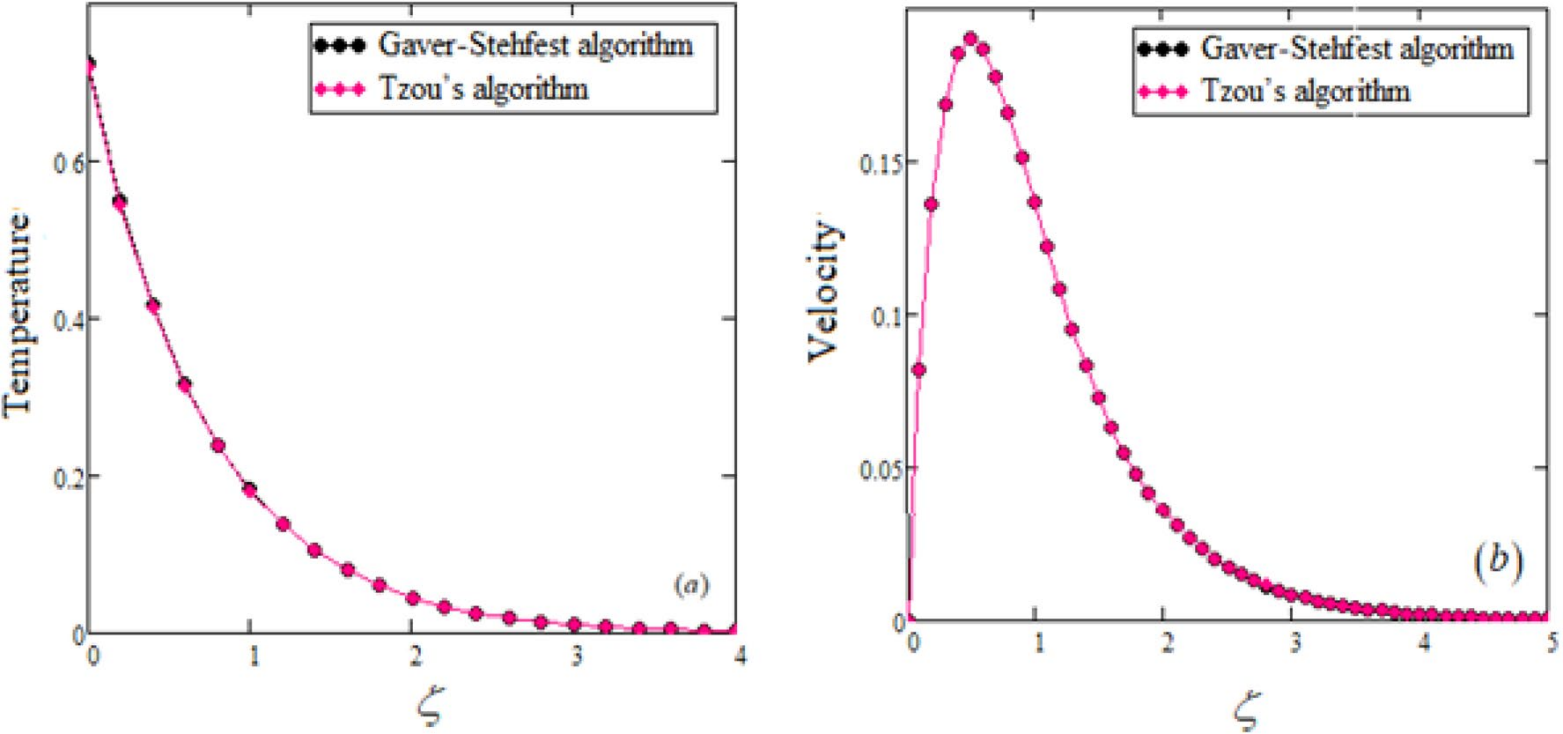
A comparable relation between temperature (**a**) and velocity (**b**) fields of our model with different numerical Laplace inversion algorithms.

This research focuses on the study of free convection of unsteady flows of an incompressible MHD Jaffrey-type fluid over an boundless movable upright flat plate with uniform heat flux. The study uses Prabhakar fractional derivative in the constitutive equations to consider generalized memory effects. The Laplace transform technique derives dimensionless temperature and velocity fields regarding exponential functions. The solutions of the conventional model are also obtained as a special case corresponding to the integer order derivative. Numerical results are gained using Mathcad software, and the outcomes are presented in graphical form in Figs. 1, 2, 3, 4, and 5. The comprehensive result of the problem with fractional derivatives is compared with classical fluids using two numerical inversion slants, namely Gaver–Stehfest’s and Tzou’s, in tabular and graphical forms.

All parameters and profiles used in this study are dimensionless. The effects of fractional parameters, Isuch as $\alpha ,\,\,\beta$ and $\gamma$, on the thermal and fluid velocity profiles, were investigated. The influence of the Prandtl number $\Pr ,$ absorption parameter $Q_{0}$, thermal Grashof number $Gr$, magnetic parameters $M,$ Jeffrey parameters $\lambda$, $\lambda_{1}$ and time $t$ on velocity profiles was also explored. The correlation between the fractional Jeffrey fluid and the fractional viscous fluid and the ordinary Jeffrey fluid and the ordinary viscous fluid was investigated. The impact of the fractional parameters $\alpha ,\,\,\beta$ and $\gamma$ on the thermal and momentum profiles while keeping the other parameters constant is shown in Figs. 2a–c and 3a–c. The thermal and momentum profiles increase as the values of fractional parameters $\alpha$ and $\beta ,$ increase, while they decline as the values of fractional parameters increase $\gamma .$

**Figure 6:**
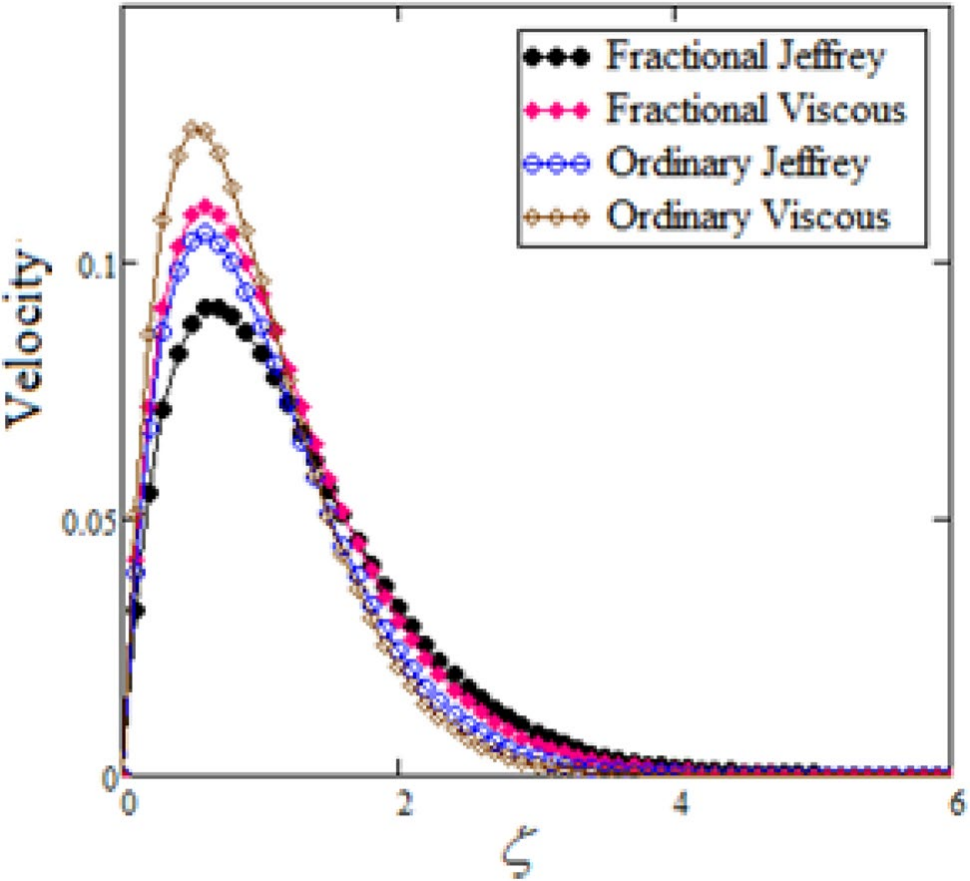
Comparison between ordinary and fractional fluids.

Figures 2 and 3 depict the effects of numerous parameters on a flowing fluid’s temperature and velocity profiles. Specifically, Fig. 2d,e show the impact of fractional parameters $\Pr$ and $Q_{0}$ on the temperature profile, which indicates a decay behavior for $\Pr$ and $Q_{0}$. As the value increases, the fluid's thermal conductivity decreases, and the viscosity increases, leading to a decrease in temperature. Additionally, as time increases, the temperature field decreases, as shown in Fig. 2f.

On the other hand, Fig. 3d,e illustrate the impact of heat absorption $Q_{0}$ and the Prandtl number $\Pr$ on the velocity profile. As the value of $\Pr$ increases, the velocity profile displays a decaying behavior due to increased fluid viscosity and decreased thermal conductivity.

Furthermore, Fig. 3 shows the fluctuation of velocity distribution caused by the Grashof number $Gr.$ As the value of $Gr$ increases, the buoyancy effect grows, which affects the thickness of the fluid shear boundary, and the fluid velocity profile rises to its maximum height close to the plate.

Finally, Fig. 3g evaluates the influence of the magnetic flux on the momentum profile, which decreases as the magnetic parameter $M,$ increases. Physically, changes in the magnetic field increase the Lorentz force, decreasing the momentum field.

Figure 3h–i depict the effects of the Jeffrey parameters $\lambda$ and $\lambda_{1}$ on fluid velocity, showing that the velocity decreases with an increase in the Jeffrey fluid parameter $\lambda ,$ while it rises with an increase in the Jeffrey parameter $\lambda_{1}.$ Figure 3j shows the influence of time on fluid motion, indicating that the fluid motion increases with time $t.$

To validate our gotten results for temperature and velocity outlines, Fig. 4a,b compare them with previously published work.

The reliability of various numerical inversion methods for the Laplace transform is demonstrated in Table 1 and Fig. 5a,b, which show perfect agreement.

**Table 1:** Model validation for different values of fractional parameters.

Fractional parameters	Temperature with Stehfest’s	Temperature with Tzou’s	Velocity with Stehfest’s	Velocity with Tzou’s
0.0	0.724	0.718	0	0
0.2	0.55	0.544	0.136	0.136
0.4	0.417	0.413	0.186	0.186
0.6	0.316	0.314	0.187	0.187
0.8	0.24	0.239	0.166	0.166
1	0.182	0.181	0.137	0.137
1.2	0.138	0.138	0.108	0.108
1.4	0.105	0.105	0.083	0.083
1.6	0.08	0.079	0.063	0.063
1.8	0.06	0.06	0.048	0.048
2	0.046	0.046	0.036	0.036
2.2	0.035	0.035	0.027	0.027
2.4	0.026	0.026	0.02	0.02
2.6	0.015	0.02	0.015	0.015
2.8	0.011	0.015	0.011	0.011
3	0.00866	0.011	0.008603	0.008604

Figure 6 compares the fractional Jeffery and viscous fluid models with ordinary Jeffery and viscous fluid models, revealing that viscous fluids flow faster than Jeffery fluids in ordinary and fractional cases. The velocity field exhibits the same behavior in both classical and fractional models. Notably, the velocity of an ordinary Jeffery fluid or a viscous fluid is greater than that of a fractional Jeffery fluid or a viscous fluid.

## Conclusions

In this article, we have considered the free convection of unsteady flows of an incompressible MHD Jaffrey-type fluid flows over an boundless movable vertical flat plate with uniform heat flux. The time-fractional Prabhakar derivative is used in the constitutive equation to generalize the memory effect. The Laplace transform procedure is used to attain the precise solution for both momentum and thermal profiles. A comparison of fractional viscous fluid and fractional Jeffrey fluid, as well as an ordinary viscous fluid and an ordinary Jeffrey fluid, is also given by using. Using the Prabhakar operators with certain fractional coefficient values could be a good way to choose a mathematical model that fits both experimental and theoretical data. The following are some important conclusions:

- The velocity profile of fractional Jeffrey fluid increases by increasing the values of fractional parameters $\alpha$ and $\beta ,$ while the reverse behavior is seen for the fractional parameter $\gamma$.
- It can be observed that when $Q_{0}$ and $\Pr$ are boosted, the fluid’s velocity and temperature graphs drop.
- With larger values of Grashof numbers $Gr$, the fluid velocity increases. A reversed effect is examined for $M$.
- It is investigated whether the velocity field is elevated as time values increase.
- Enhancing the Jeffrey parameter $\lambda$ reduces the fluid velocity, while an opposite effect is observed for $\lambda_{1}$.
- It can be seen that fractional Jeffrey fluid and fractional viscous fluid move more slowly than ordinary Jeffrey fluid and ordinary viscous fluid.
- Our required solutions are equivalent due to inversion procedures, namely Stehfest's and Tzou.

## Supplementary Information

Supplementary Information.

## Data Availability

The data used to support the findings of this study are available from the corresponding author upon request.

## List of symbols

$$x,y$$: Cartesian coordinates
$v(y,t)$: Velocity of the fluid (ms^−^^1^)
$\theta (y,t)$: Temperature of the fluid (K)
$\theta_{\infty } (y,t)$: Fluid temperature far away from the plate (K)
$Q_{0}$: Heat absorption coefficient (Wm^−^^3^ K^−^^1^)
$c_{p}$: Specific heat at constant pressure (Wm^−^^1^ K^−^^1^)
$Gr$: Thermal Grashof number
$$\Pr$$: Prandtl number
$$M$$: Magnetic parameter
$k$: Fluid thermal conductivity (Wm^−^^1^ K^−^^1^)
$$g$$: Gravitational acceleration (m s^−^^2^)
$q$: Thermal flux (Wm^−^^2^)
$q_{0}$: Constant heat flux per unit area at the plate
$$t$$: Time (s)

## Greek symbols

$$\alpha ,\,\,\beta ,\,\,\gamma$$: Fractional parameters
$\lambda_{1}$: Jeffrey’s fluid parameter
$$\lambda$$: Retardation time (s)
$\rho$: Fluid density (kgm^−^^3^)
$\beta_{1}$: Thermal expansion coefficient (k^−^^1^)
$\sigma$: Electrical conductivity (sm^−^^1^)
$$\nu$$: Kinematic viscosity (m^2^s^−^^1^)
$\mu$: Dynamic viscosity (kgm^−^^1^ s^−^^1^)
