# Supplementary material for: MHD Free convection flows of Jeffrey fluid with Prabhakar-like fractional model subject to generalized thermal transport
Source: Sci Rep. 2023 Jun 7;13:9289. doi: 10.1038/s41598-023-36436-2 (PMC10247771; doi:10.1038/s41598-023-36436-2)
Supplement: Supplementary file 1 — Supplementary Information. [file 41598_2023_36436_MOESM1_ESM.docx]

**Appendix A**

$\begin{matrix} L^{-1}\left\{\frac{e^{-y\sqrt{\frac{as+b}{cs+1}}}}{s}\right\}=\frac{a}{c}e^{-\frac{t}{c}}\int_{0}^{\infty} e^{-\frac{au}{c}}erfc\left(\frac{y}{2\sqrt{u}}\right)I_{0}\left(\frac{2}{c}\sqrt{\left(a-bc\right)ut}\right)du \\ +\frac{b}{c}\int_{0}^{\infty} \int_{0}^{t} e^{-\frac{au+s}{c}}erfc\left(\frac{y}{2\sqrt{u}}\right)I_{0}\left(\frac{2}{c}\sqrt{\left(a-bc\right)us}\right)dsdu. \end{matrix}$ (A.1)

$L^{-1}\left\{\frac{e^{-a\sqrt{s+b}}}{s}\right\}=\frac{1}{2}\left\{e^{a\sqrt{b}}erfc\left(\frac{a}{2\sqrt{t}}+\sqrt{bt}\right)+e^{-a\sqrt{b}}erfc\left(\frac{a}{2\sqrt{t}}-\sqrt{bt}\right)\right\}$ (A.2)
